# Associations of a *PTPN11 *G/A polymorphism at intron 3 with *Helicobactor pylori *seropositivity, gastric atrophy and gastric cancer in Japanese

**DOI:** 10.1186/1471-230X-9-51

**Published:** 2009-07-09

**Authors:** Asahi Hishida, Keitaro Matsuo, Yasuyuki Goto, Mariko Naito, Kenji Wakai, Kazuo Tajima, Nobuyuki Hamajima

**Affiliations:** 1 Department of Preventive Medicine/Biostatistics and Medical Decision Making, Nagoya University Graduate School of Medicine, Nagoya, Japan; 2 Division of Epidemiology and Prevention, Aichi Cancer Center Research Institute, Nagoya, Japan

## Abstract

**Background:**

Previous studies have revealed the significance of *Helicobacter pylori *(*H. pylori*) infection as a risk factor of gastric cancer. Cytotoxin-associated gene A (*cagA*) positivity has been demonstrated to determine the clinical outcome of *H. pylori *infection in the presence of SHP-2 (src homology 2 domain-containing protein tyrosine phosphatase-2). This study aimed to examine the formerly reported association of G/A *PTPN11 (protein-tyrosine phosphatase, nonreceptor-type 11) *polymorphism (rs2301756) with gastric atrophy, as well as the association with gastric cancer in a Japanese population using a large sample size.

**Methods:**

Study subjects were 583 histologically diagnosed patients with gastric cancer (429 males and 154 females) and age- and sex-frequency-matched 1,636 non-cancer outpatients (1,203 males and 433 females), who visited Aichi Cancer Center Hospital between 2001–2005. Serum anti-*H. pylori *IgG antibody and pepsinogens were measured to evaluate *H. pylori *infection and gastric atrophy, respectively. Odds ratios (ORs) and 95% confidence intervals (CIs) were calculated by a logistic model.

**Results:**

Among *H. pylori *seropositive non-cancer outpatients, the age- and sex-adjusted OR of gastric atrophy was 0.82 (95% CI 0.62–1.10, *P *= 0.194) for *G/A*, 0.84 (95% CI 0.39–1.81, *P *= 0.650) for *A/A*, and 0.83 (95% CI 0.62–1.09, *P *= 0.182) for *G/A*+*A/A*, relative to *G/G *genotype, and that of severe gastric atrophy was 0.70 (95% CI 0.47–1.04, *P *= 0.079), 0.56 (95% CI 0.17–1.91, *P *= 0.356), and 0.68 (95% CI 0.46–1.01, *P *= 0.057), respectively. Among *H. pylori *infected subjects (*H. pylori *seropositive subjects and seronegative subjects with gastric atrophy), the adjusted OR of severe gastric atrophy was further reduced; 0.62 (95% CI 0.42–0.90, *P *= 0.012) for *G/A*+*A/A*. The distribution of the genotype in patients with gastric cancer was not significantly different from that for *H. pylori *infected subjects without gastric atrophy.

**Conclusion:**

Our study results revealed that those with the *A/A *genotype of *PTPN11 *rs2301756 polymorphism are at lower risk of severe gastric atrophy, but are not associated with a decreased risk of gastric cancer, which partially supported our previous finding that the polymorphism in the *PTPN11 *gene encoding SHP-2 was associated with the gastric atrophy risk in *H. pylori *infected Japanese. The biological roles of this *PTPN11 *polymorphism require further investigation.

## Background

*Helicobacter pylori *(*H. pylori*) infection is a well-established risk factor of gastric cancer through the development of gastric atrophy and subsequent precancerous lesions. Particularly, *H. pylori *strains with the cytotoxin-associated gene A (*cagA*) are in a strong association with increased gastric adenocarcinoma risk [1]. Severe gastric atrophy and corpus-predominant gastritis along with intestinal metaplasia are well established as predominant predispositions to gastric cancer [2]. Host proinflammatory genetic factors in combination with bacterial virulence factors such as CagA have been reported to determine the severity of gastric damage and the eventual clinical outcome of *H. pylori *infection [3,4]. The risk of gastric cancer is multiplied several fold if the host harbors both of these factors [5,6]. In East Asian populations, great majority of *H. pylori *are *cagA*-positive strains. CagA is divided into two major subtypes, East Asian type and Western type [7]. The grade of gastric atrophy and gastric cancer risk is higher in patients with East Asian *cagA*-positive strains than in those with *cagA*-negative or Western *cagA*-positive strains [8].

CagA protein is translocated from attached *H. pylori *into host gastric epithelial cells via a bacterial type IV secretion apparatus, and undergoes tyrosine phosphorylation in the host cells [9]. It induces the scattering phenotypes in gastric epithelial cells, called the "hummingbird phenotype," which is thought to play a crucial role in the pathogenesis of *cagA*-positive *H. pylori *infection, eventually leading to gastric carcinoma. In this CagA-dependent morphological transformation of gastric epithelial cells, the existence of SHP-2 (src homology 2 domain-containing protein tyrosine phosphatase-2) is essential [10]. SHP-2 plays a key role in intracellular signaling downstream of a number of growth factors, hormones, and cytokines [11,12]. The translocated CagA forms a physical complex with SHP-2 thereby stimulating its phosphatase activity [10]. Subsequent Erk (extracellular signal-regulated kinase) activity also contributes to the CagA-induced "hummingbird phenotype" [13]. Thus, CagA/SHP-2 complex formation may induce abnormal proliferation, movement of gastric epithelial cells and cellular changes that might conclusively lead to gastric atrophy and gastric carcinoma.

**Figure 1:**
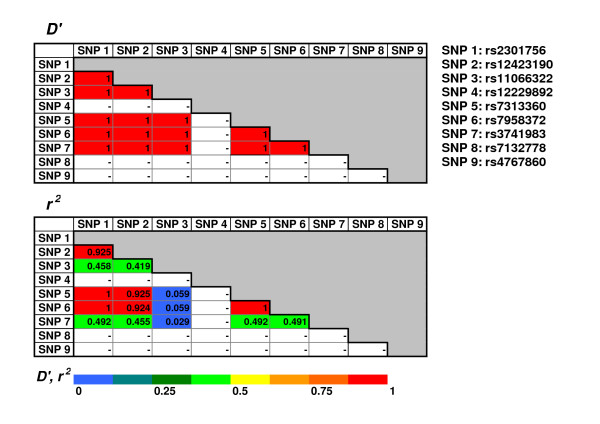
**Linkage disequilibrium (LD) between the 9 single nucleotide polymorphisms (SNPs) with a minor allele frequency > 0.05 in the *PTPN11 *gene region among Caucasians (CEU: Utah residents with ancestry from northern and western Europe)**. LD maps are shown by two parameters, *r*^2 ^and *D*' for Caucasians (CEU: Utah residents with ancestry from northern and western Europe). SNP numbers in the LD maps correspond to the rs numbers described in the upper right of the maps.

Since SHP-2 closely interacts with the CagA protein, it is natural to speculate that functional polymorphisms in the *PTPN11 (protein-tyrosine phosphatase, nonreceptor-type 11) *gene encoding SHP-2 may ultimately influence the degree of gastric atrophy and transformation to gastric cancer in infected subjects. There are 9 single nucleotide polymorphisms (SNPs) at minor allele frequency > 0.05 in *PTPN11 *gene in Japanese on HapMap, all of which are located in non-coding regions, and most of them are in absolute linkage disequilibrium (LD) (*D' *= 1 and *r*^2 ^= 1) or complete linkage disequilibrium (*D' *= 1 and *r*^2 ^< 1) to each other. Five of the 9 SNPs are shown to be in complete LD and 3 of them are shown to be in absolute LD or nearly absolute LD (*D' *= 1 and *r*^2 ^> 0.9) in Caucasians as shown in Figure 1, based on HapMap homepage http://www.hapmap.org. Our recent report revealed that one *PTPN11 G/A *SNP at intron 3 (rs2301756) was in complete LD to another *PTPN11 G/A *SNP at intron 10 (rs12229892) [14]. In this study, the *G/A *SNP at intron 3 (rs2301756), one of the 3 SNPs in absolute LD was selected as a representative of these linked SNPs.

In *H. pylori *related gastric cancer, the process leading to disease has three steps; *H. pylori *infection, gastric atrophy development and carcinogenesis. At each step, genetic traits and their interactions with lifestyle may influence the process [15]. For genetic traits, significant associations of *interleukin *(*IL*) *-1B *C-31T and C-511T polymorphisms [16,17], *tumor necrosis factor (TNF)-α *C-857T and T-1031C polymorphisms [18], and a NAD(P)H dehydrogenase, quinone 1 *(NQO1) *C609T polymorphism [19] with *H. pylori *infection, and associations of a *G/A *polymorphism at intron 2 of *Grb2-associated binder 1 *(*Gab1*) [20], *interleukin *(*IL*) 2 T-330G, and *IL-13 *C-1111T [21] with gastric atrophy have been reported to date. Variable number of tandem repeats (VNTR) polymorphisms of *mucin 1 (MUC1) *have also been shown to influence *H. pylori *infection [22,23]. Although there are several studies to demonstrate the polymorphisms significantly associated with the risk of gastric cancer, only a few studies evaluated the risk for the step from gastric atrophy to gastric cancer [15].

In our previous studies among Japanese and Japanese Brazilians, the *AA *genotype in intron 3 was shown to reduce the risk of gastric atrophy development [24,25]. Recently, another *PTPN11 *SNP (rs11066322 at intron 10 in complete linkage disequilibrium to rs2301756) was found to be significantly associated with serum apoB levels in a British population [26], supporting the hypothesis that this *PTPN11 *polymorphism is functional. This study aimed to confirm the formerly reported association between the *PTPN11 *polymorphism (rs2301756) and gastric atrophy measured with serum pepsinogens in a large number of Japanese subjects, as well as to examine the association with gastric cancer risk.

## Methods

### Subjects

Subjects were participants of HERPACC (Hospital-based Epidemiologic Research Program at Aichi Cancer Center) study, in which first-visit outpatients were consecutively invited to provide lifestyle data and blood sample after obtaining informed consent [27]. Among the participants who visited Aichi Cancer Center Hospital from 2001 – 2005, 583 cases diagnosed as gastric cancer and age- and sex-frequency-matched 1,638 cancer free outpatients were sampled as a control group, among whom two outpatients were excluded because they could not be genotyped, leaving 583 cases and 1,636 controls eligible for the analyses. Informed consent was obtained from all the subjects and the study protocol was approved by the Ethics Committees of Aichi Cancer Center and Nagoya University Graduate School of Medicine.

### Samples and diagnostic criteria

Their serum samples were immediately stored at -20°C until analysis. Anti-*H. pylori *IgG antibody was measured with an enzyme immunoassay (EIA) kit "E plate 'Eiken' *H. pylori *Antibody" (Eiken Kagaku, Tokyo, Japan). According to the instructions provided with this kit, 10.0 units or higher was regarded as seropositive. Serum pepsinogens (PG) were measured by chemiluminescence enzyme immunoassay (CLEIA). Gastric mucosal atrophy was grouped into "none" (PG I > 70 ng/ml or PG I/PG II > 3), "mild" (PG I ≤ 70 ng/ml and PG I/PG II ≤ 3, excluding "severe" cases), or "severe" (PG I ≤ 30 ng/ml and PG I/PG II ≤ 2). Since serum samples of gastric cancer cases were planned to be used for a study with higher priority, the antibody and PGs of the cases were not measured.

### Genotyping

DNA was extracted from buffy coat using the Qiagen DNeasy mini kit (Qiagen, Hilden, Germany). The *PTPN11 *G/A polymorphism (rs2301756) was genotyped with a polymerase chain reaction with confronting two-pair primers (PCR-CTPP) [28]. The primers were F1: GGA TTA CAG GCA TAA GCC AC, R1: GAC CAC TAA ACT TCT TAA ATG AGC, F2: CAT TTG TCT CTA AAG GAC TGT GGA, and R2: CTC TGG CTC TCT CGT ACA AGA. Amplification conditions were 10 min of initial denaturation at 95°C, followed by 30 cycles of 1 min at 95°C, 1 min at 64°C, and 1 min at 72°C, then a 5-min final extension at 72°C. The amplified DNA was visualized on a 2% agarose gel with ethidium bromide staining. The amplified DNA was 201 bp for *G/G *genotype, 201 bp and 339 bp for *G/A *genotype, 339 bp for *A/A *genotype, and 490 bp for common band [24].

### Statistical analysis

The differences in proportions were examined with a Fisher's exact test. The 95% confidence intervals (CIs) for percentages were calculated based on binomial distributions. Logistic regression analysis was performed for estimating odds ratios (ORs) and 95% CIs. Age was adjusted as a continuous variable in the logistic model. *H. pylori *infected subjects was defined as those with *H. pylori *seropositivity or with gastric atrophy, because in the great majority of cases gastric atrophy develops after *H. pylori *infections. The trends for *H. pylori *infection, gastric atrophy or gastric cancer development by sex or age categories were compared using the χ^2 ^test for trend. The calculations were done using the STATA version 7 (Stata Corp, College Station, TX).

## Results

### Characteristics of the subjects and allele frequency of the PTPN11 polymorphism

The characteristics of the subjects are summarized in Table 1. The mean age ± standard deviation was 58.7 ± 10.6 y (range: 25–84 y) for controls and 58.8 ± 10.5 y (range: 27–80 y) for cases. Females were 26.5% in controls and 26.4% in cases. About three quarters of the controls were infected with *H. pylori*, while about one third of the controls had gastric atrophy. The genotype frequency of the *PTPN11 *polymorphism among the controls was in Hardy-Weinberg's equilibrium (χ^2 ^= 0.047, *P *= 0.828). We tested the trend for *H. pylori *infection, gastric atrophy or gastric cancer development by sex or age categories, which revealed significant trend for higher *H. pylori *infection rate in males (*P*-value for trend < 0.001) and higher age categories (*P *< 0.001), and for higher prevalence of gastric atrophy in higher age categories (*P *< 0.001).

**Table 1:** Characteristics of the subjects and the *PTPN11 *rs2301756 polymorphism.

	Controls *n *= 1636			Cases *n *= 583
	***H. pylori (-)***	***H. pylori (+)***		
		**GA (-)**	**GA (+)**	
***n***	699	442	495	583
				
**Sex**				
Male	479 (68.5%)	363 (82.1%)	361 (72.9%)	429 (73.6%)
Female	220 (31.5%)	79 (17.9%)	134 (27.1%)	154 (26.4%)
				
**Age**				
< 30	6 (0.9%)	1 (0.2%)	1 (0.2%)	2 (0.3%)
30–39	67 (9.6%)	11 (2.5%)	3 (0.6%)	31 (5.3%)
40–49	138 (19.7%)	54 (12.2%)	31 (6.3%)	64 (11.0%)
50–59	194 (27.8%)	151 (34.2%)	141 (28.5%)	214 (36.7%)
60–69	211 (30.2%)	152 (34.4%)	221 (44.7%)	166 (28.5%)
70-	83 (11.9%)	73 (16.5%)	98 (19.8%)	106 (18.2%)
				
**Genotype**				
*G*/*G*	483 (69.1%)	293 (66.3%)	350 (70.7%)	396 (67.9%)
*G*/*A*	198 (28.3%)	135 (30.5%)	131 (26.5%)	174 (29.9%)
*A*/*A*	18 (2.6%)	14 (3.2%)	14 (2.8%)	13 (2.2%)

GA(-) and GA(+) indicate without atrophy and with atrophy, respectively.

### PTPN11 polymorphism, *H. pylori *seropositivity, gastric atrophy and gastric cancer

There was no significant association between the *PTPN11 *polymorphism and the seropositivity, although the OR of *A/A *genotype was 1.19 relative to *G/G *genotype (Table 2).

**Table 2:** Odds ratios (ORs) and 95% confidence intervals (CIs) of *PTPN11 *rs2301756 polymorphism for *H. pylori *seropositivity.

Genotype, allele	*n*	*H. pylori *+	*H. pylori *+ (%)	OR^a^	95% CI	*P *value
*G*/*G*	1126	643	57.1	1	Reference	-
*G*/*A*	464	266	57.3	1.02	0.81–1.28	0.865
*A*/*A*	46	28	60.9	1.19	0.64–2.22	0.577
						
*G*	2716	1552	57.1	1	Reference	-
*A*	556	322	57.9	1.03	0.85–1.25	0.387

^a^OR for each genotype was calculated by age and sex adjusted logistic regression model, and a crude OR was calculated for each allele.

There were 937 *H. pylori *seropositive subjects, among whom 495 (52.8%) subjects had atrophy. On the one hand, there were 45 (6.4%) subjects with atrophy among 699 seronegative subjects. The difference in the prevalence was statistically significant (*P *< 0.001).

Table 3 shows the genotype distribution according to seropositivity and atrophy. There were no subjects with *A/A *genotype among the seronegative atrophy participants. Accordingly, the adjusted OR of gastric atrophy among the seronegative subjects was not calculable for the *A/A *genotype; the genotype distribution was not significantly associated with gastric atrophy by a 3 × 3 Fisher's exact test (*P *= 0.196).

**Table 3:** *PTPN11 *rs2301756 genotype distribution according to *H. pylori *seropositivity and the grade of gastric atrophy.

Genotype	***H. pylori *seronegative**			***H. pylori *seropositive**		
	GA (-)	GA (+)	GA (++)	GA (-)	GA (+)	GA (++)
*G*/*G*	447 (68.3%)	11 (64.7%)	25 (89.3%)	293 (68.4%)	223 (68.2%)	127 (75.6%)
*G*/*A*	189 (28.9%)	6 (35.3%)	3 (10.7%)	135 (28.9%)	93 (28.4%)	38 (22.6%)
*A*/*A*	18 (2.8%)	0 (0.0%)	0 (0.0%)	14 (2.7%)	11 (3.4%)	3 (1.8%)
Total	654 (100%)	17 (100%)	28 (100%)	442 (100%)	327 (100%)	168 (100%)

GA(-), GA(+) and GA(++) indicate no atrophy, mild atrophy and severe atrophy, respectively.

The age- and sex-adjusted OR of gastric atrophy among *H. pylori *seropositive subjects was 0.82 (95% CI 0.62–1.10, *P *= 0.194) for *G/A*, 0.84 (95% CI 0.39–1.81, *P *= 0.650) for *A/A*, and 0.83 (95% CI 0.62–1.09, *P *= 0.182) for *G/A*+*A/A*, compared with *G/G *genotype. The age- and sex-adjusted OR of severe gastric atrophy among *H. pylori *seropositive subjects was 0.70 (95% CI 0.47–1.04, *P *= 0.079) for *G/A*, 0.56 (95% CI 0.17–1.91, *P *= 0.356) for *A/A*, and 0.68 (95% CI 0.46–1.01, *P *= 0.057) for *G/A*+*A/A *when those without severe gastric atrophy were defined as a reference (Table 4). When *H. pylori *seropositive subjects and seronegative subjects with gastric atrophy were regarded as *H. pylori *infected subjects, the age- and sex-adjusted OR of severe gastric atrophy among the *H. pylori *infected was further reduced; 0.62 (95% CI 0.42–0.90, *P *= 0.012) for *G/A*+*A/A *(Table 4).

**Table 4:** Genotype frequencies for *PTPN11 *rs2301756 polymorphism, odds ratios (ORs) and 95% confidence intervals (CIs) of gastric atrophy in the *H. pylori *seropositive subjects (a) and *H.pylori *infected subjects (b)

(a)									
**Genotype, allele**	***n***	**All gastric atrophy (%)**	**OR^a^**	**95% CI**	***P *value^b^**	**Severe gastric atrophy (%)**	**OR^b^**	**95% CI**	***P *value^b^**

*G*/*G*	643	350 (54.4)	1	Reference	-	127 (19.8)	1	Reference	-
*G*/*A*	266	131 (49.2)	0.82	0.62–1.10	0.194	38 (14.3)	0.70	0.47–1.04	0.079
*A*/*A*	28	14 (50.0)	0.84	0.39–1.81	0.650	3 (10.7)	0.56	0.17–1.91	0.356
*G*/*A*+*A*/*A*	294	145 (49.3)	0.83	0.62–1.09	0.182	41 (13.9)	0.68	0.46–1.01	0.057
									
*G*	1552	831 (53.5)	1	Reference	-	292 (18.8)	1	Reference	-
*A*	322	159 (49.4)	0.85	0.66–1.08	0.097	44 (13.7)	0.67	0.45–0.96	0.015

**(b)**									

**Genotype, allele**	***n***	**All gastric atrophy (%)**	**OR^a^**	**95% CI**	***P *value^b^**	**Severe gastric atrophy (%)**	**OR^a^**	**95% CI**	***P *value^b^**

*G*/*G*	677	383 (56.6)	1	Reference	-	151 (22.3)	1	Reference	-
*G*/*A*	274	139 (50.7)	0.80	0.60–1.06	0.123	41 (15.0)	0.63	0.43–0.93	0.019
*A*/*A*	28	14 (50.0)	0.77	0.36–1.67	0.512	3 (10.7)	0.49	0.14–1.67	0.254
*G*/*A*+*A*/*A*	302	153 (50.7)	0.80	0.60–1.05	0.106	44 (14.6)	0.62	0.42–0.90	0.012
*G*	1628	905 (55.6)	1	Reference	-	343 (21.1)	1	Reference	-
*A*	330	167 (50.6)	0.82	0.64–1.05	0.102	47 (14.2)	0.62	0.44–0.87	0.005

^a^OR for each genotype was calculated by age and sex adjusted logistic regression model, and a crude OR was calculated for each allele.

^b^*P *values less than 0.05 are shown in *Italics*.

**Figure 2:**
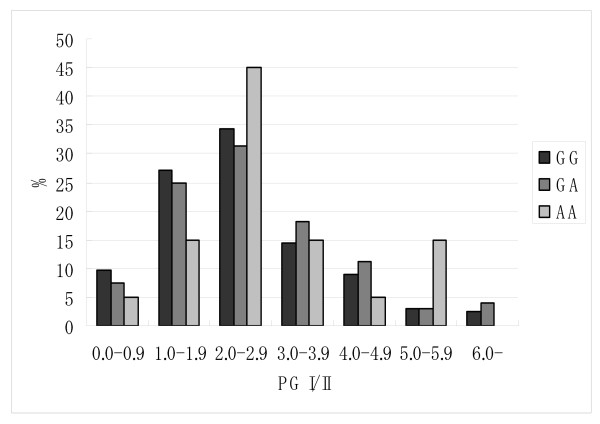
**Distributions of pepsinogen (PG) I/II ratio according to *PTPN11 *rs2301756 genotype among *H. pylori *infected subjects with a PG I level of ≤ 70 ng/ml**.

Figure 2 depicts the distributions of pepsinogen I/II ratio according to *PTPN11 *rs2301756 genotype among *H. pylori *infected subjects with PG I ≤ 70 ng/ml. In accordance with the finding that subjects with *A *allele were at significantly reduced risk of severe gastric atrophy, the frequency of subjects with PG I/II ratio less than 2 was lower in subjects with *A *allele than those with *G/G *genotype.

To investigate how this polymorphism of *PTPN11 *contributes to the gastric carcinogenesis among the *H. pylori *infected subjects, we also calculated the OR of gastric cancer compared with *H. pylori *infected subjects without gastric atrophy. The age- and sex-adjusted OR of gastric cancer was 0.97 (95% CI 0.74–1.28, *P *= 0.839) for *G/A*, 0.71 (95% CI 0.33–1.53, *P *= 0.381) for *A/A*, and 0.95 (95% CI 0.73–1.23, *P *= 0.689) for *G/A*+*A/A*, relative to *G/G *genotype, none of which were statistically significant (Table 5).

**Table 5:** Genotype frequencies for *PTPN11 *rs2301756 polymorphism, age-sex adjusted odds ratios (ORs) and 95% confidence intervals (CIs) of gastric cancer relative to *H. pylori *infected subjects without gastric atrophy (HP-infected without atrophy).

Genotype	HP infected without atrophy *n *= 442	Gastric cancer *n *= 583	OR	95% CI	*P value*
*G*/*G*	293 (66.3%)	396 (67.9%)	1	Reference	-
*G*/*A*	135 (30.5%)	174 (29.8%)	0.97	0.74–1.28	0.839
*A*/*A*	14 (3.2%)	13 (2.2%)	0.71	0.33–1.53	0.381
*G*/*A*+*A*/*A*	149 (33.7%)	187 (32.1%)	0.95	0.73–1.23	0.689
					
*G*	721 (81.6%)	966 (82.8%)	1	Reference	-
*A*	163 (18.4%)	200 (17.2%)	0.92	0.72–1.16	0.243

^a^OR for each genotype was calculated by age and sex adjusted logistic regression model, and a crude OR was calculated for each allele.

## Discussion

This study revealed that those who harbor *A *allele of the *PTPN11 *rs2301756 polymorphism at intron 3 had a significantly lower risk of severe gastric atrophy. This is in accordance with our hypothesis that gastric atrophy development after *cagA*-positive *H. pylori *infection was rarer among those with *A/A *genotype than among those with *G/G *genotype [24,25], although the significant association was observed only for severe gastric atrophy in this study. Since the biological processes in infection, atrophy development, and carcinogenesis are different [15], the association of this polymorphism with only atrophic development seemed biologically plausible.

While there was limited information on the potential function of *PTPN11 *polymorphisms, biological characteristics of SHP-2 have become increasingly understood. SHP-2 is one of the two existing mammalian non-transmembrane (intracellular) protein tyrosine phosphatases (PTPs) that contain src homology 2 (SH2) domains. Binding of tyrosine phosphorylated CagA to the SH2 domains is supposed to induce conformational change in SHP-2 that weakens the inhibitory interaction between PTP and N-terminal SH2 domains, eventually leading to the activation of SHP-2 phosphatase [10,29,30]. The *G/A *polymorphism in the intron 3 of *PTPN11 *is located 223 base pairs upstream from exon 4, which encodes the initial part of C-terminal SH2 domain. Although the biological role of the present polymorphism is not yet clearified, the polymorphism may have some influence on the formation of *PTPN11 *mRNA splicing variants, of which 8 forms have been reported to date (SpliceMinor homepage developed by The Genomics & Bioinformatics Group (GBG) of NCI: http://www.tigerteamconsulting.com/SpliceMiner/). The LD data between the *PTPN11 *SNPs shown in Figure 1 is that for Caucasians, and no precise information about LD in Japanese is available at present. There are also some *PTPN11 *SNPs whose LD status is left unknown (SNP8 and SNP9 in Figure 1). The function of the *PTPN11 *polymorphism at exon 3 (rs2301756) and other *PTPN11 *SNPs on the interaction between SHP-2 and CagA in Japanese and other ethnicities requires further investigation.

Although the *G *allele of *PTPN11 *may be a part of the genetic traits to develop gastric atrophy via signal transduction from CagA, there seems to be other genetic traits involved in this process. CagA binds several molecules; Grb2, which transduces the signal to Ras-MAPK pathway causing cell proliferation, c-Met hepatocyte growth factor (HGF) receptor, which have a role of cell proliferation and motility, ZO-1, a tight-junction protein, and Par1/Mark kinase, which has an essential role in epithelial cell polarity [31-35]. Although no studies have been conducted, functional polymorphisms of these molecules might be also candidates of genetic traits of gastric atrophy.

The *A *allele was the dominant allele of *PTPN11 *polymorphism at intron 3 among Caucasians (0.875 of 120 chromosomes), but not among Japanese (0.178 of 902 chromosomes) and Chinese (0.083 of 48 choromosomes) [26]. This study found that the *A *allele frequency was 0.170 among our Japanese control subjects, similar to the reported allele frequency in Japanese.

The present study has several limitations. Although the *H. pylori *status of the control subjects was examined with a serology test, we did not check the CagA status. As reported in Japan, nearly 100% of *H. pylori *strains possess a functional *cag *pathogenicity island (*cag *PAI), which encodes and produces the CagA protein [36]. A previous study also certified that almost all strains isolated from our Japanese subjects were East Asian *cagA*-positive strains [37], indicating that *H. pylori *strains in our study subjects also possesses CagA. Another limitation is related to the diagnosis of gastric atrophy. This was done entirely on the basis of serum pepsinogen levels and not through histological assessment, because most of the control subjects did not undergo gastrointestinal endoscopy with biopsy. However, the pepsinogen method is well established as a surrogate marker of gastric atrophy [38-40]. The validated criterion for gastric atrophy is PG I ≤ 70 ng/ml and PG I/II ratio of ≤ 3.0, and that for severe gastric atrophy is PG I ≤ 30 ng/ml and PG I/II ratio of ≤ 2.0, both of which are supposed to be reliable because they are widely used in practice in Japan [41,42]. Concerning the gastric cancer cases the *H. pylori *seropositivity and pepsinogen levels were not examined, but most of the gastric cancer cases seemed to be *H. pylori *positive cases with gastric atrophy [43,44]. Considering that intestinal type of gastric cancer, the predominant type of gastric cancer in Japan, arises from gastric atrophy caused by *H. pylori *infection, and diffuse type gastric cancer occurs regardless of gastric atrophy [45], it would seem intriguing to perform the subgroup analysis according to these two histological types. This analysis might unveil the association of this *PTPN11 *polymorphism with the steps of gastric carcinogenesis more clearly. However, we could not perform this analysis because of the unavailability of the histological data. In addition, there might be several factors, including genetic, that prime the individual stomach for *H. pylori *colonization, which might modulate the effect of this *PTPN11 *polymorphism [22,23]. Further investigations with these factors taken into consideration are also expected.

## Conclusion

This study revealed that rs2301756 in the *PTPN11 *gene encoding SHP-2 increased the risk of severe gastric atrophy in *H. pylori *infected subjects in Japan. Although the significant association was limited only to severe gastric atrophy, this study partially supported the roles of this *PTPN11 *polymorphism in the gastric atrophy development. Elucidation of the function of this polymorphism will help us understand the pathogenesis of *H. pylori*-induced gastric cancer, which will lead to more effective means for the personalized prevention of gastric atrophy and subsequent gastric cancer in the near future.

## Competing interests

The authors declare that they have no competing interests.

## Authors' contributions

AH: Conducted data analysis and drafted the manuscript. KM and YG: Contributed greatly to the recruitment of the participants and data collection, and advised in preparing the manuscript. MN and KW: Contributed to data analysis, and advised in preparing the manuscript. KT and NH: Supervised study design, data collection and analysis, and advised in preparing the manuscript. All authors have given final approval of the version to be submitted.

## Pre-publication history

The pre-publication history for this paper can be accessed here:

http://www.biomedcentral.com/1471-230X/9/51/prepub
